# Annotations

**Published:** 1898-04-23

**Authors:** 


### Annotations.

### The Flash-point of Petroleum.

Some time ago we spoke of the danger involved in the low flash-point of much of the oil sold for burning in lamps. There is good oil to be had, and probably most people to whom expense is not the main consideration buy this. But it is lawful to sell oil which will flash, *i.e.*, give off explosive vapour, at a temperature of 73 degrees Fahrenheit. Oil with so low a flash-point is not permitted to be sold in most of the United States; and yet this oil is allowed to be imported into this country. The Yankees sell us that which they consider it dangerous to buy themselves. This subject was taken up by the *Star* some time ago, and then we spoke in favour of raising the flash-point. We regret, therefore, to note that the evidence laid before the Petroleum Committee which has recently been sitting opposes the raising of the flash-point of oil, and recommends instead regulations regarding the manufacture of lamps and the storage and transport of oil. That these are points over which some supervision, greater than is now exercised, might be maintained, is possibly true; but they do not touch the main question, which is, whether the present flash-point is a cause of danger or not. No one pretends that if the oil employed had a higher flash-point all the accidents which are now so prevalent would occur, although it is well known that reform in lamps as well as in oils is much required. Even Mr. Spencer, of the Control Department, London County Council, whose views as to the necessity of reforming the lamps rather than raising the flash-point are well known, says, "It is, I think, beyond question that if no oil were burned below 100 deg. Fahr. close test, lamp accidents would be far less frequent." Let us, then, have such legislation on the subject of the flash-point as is shown to be necessary, not only by the declared opinion of our own *savants*, but by the practice of the two greatest oil-producing countries, America and Russia, in neither of which is such low grade oil used.

### Sanitary Authorities and Dangerous Wells.

Attention has been called during the past week to a proceeding which, if allowed to become a precedent, may make it very difficult for sanitary authorities to close dangerous wells, and thus to prevent the dissemination of typhoid fever. It appears that at Maidstone the sanitary authority desire to close a certain well, in the immediate neighbourhood of which fourteen cases of typhoid have occurred in ten houses distributed on all sides of the spot where the well is, and in dangerous proximity to it. On analysis the water showed signs of organic impurity, not to an alarming extent when taken by themselves, but more than sufficient to prove dangerous when taken in conjunction with the known surroundings of the well. On the case coming before the magistrates, the chemists for the defence pronounced the water wholesome on chemical grounds. Their analyses, in fact, agreed with those made by the public analyst. The whole gist of the case, however, lay in a proper appreciation of the surroundings of the source from which the water was drawn. The magistrates then did a queer thing—they ordered a sample of the water to be sent to Somerset House, of all places in the world, just as if they thought it was a question of adulteration! The Somerset House people, however, did a thing queerer still, and instead of declaring at once, as they should have done, that the decision as to the safety of a given water supply was not a matter of mere chemistry, and that the affair was outside the range of their work, they sent a reply which, after giving the results of the analysis, contains the extraordinary assertion that there are "grounds for stating that the water is not liable to contamination from the immediate neighbourhood of the well," and expresses the opinion that "its use for potable purposes is not likely to prove injurious or dangerous to health." The magistrates thereupon dismissed the application, with costs against the sanitary authority. There is reason for believing that this is by no means a solitary instance of such a reference to the official chemists at Somerset House, and it is a very serious matter, for if there is one thing more certain than another it is that the safety of water for drinking purposes cannot be established by chemical analysis alone, unless, indeed, a standard of purity be insisted on which would exclude the great mass of the water of the country. If we want to know whether a sample of water is fit to drink we must take into consideration where it has come from and what processes it has undergone, as well as the results, not of one only, but of many analyses. The interference of Somerset House, a mere chemical authority, in such an important sanitary question cannot fail to be disastrous, and we are glad to find that the medical officer of health has in this case recommended his authority to apply for the intervention of the Local Government Board.

### Suicide by Gas.

The distressing suicide of a lady at Chiswick by means of gas from an ordinary stove draws attention to a mode of self-destruction which, although comparatively rare in places where the gas supplied is real coal gas, has of late become alarmingly common where so-called water-gas is used. Death is easily enough caused even by ordinary gas when an escape takes place during sleep. Coal-gas, however, is a more or less irritating substance and not very easy to breathe, and in case of attempted suicide, time is given for repentance and escape. Quite otherwise, however, is it with water-gas, highly charged as it is with carbonic oxide. This does not suffocate, but poisons. A very small quantity is sufficient to take away all power of escape, and to throw the victim into a lethargy from which there is no awakening. How many people try to poison themselves with ordinary gas but find it nasty we do not know—no one knows. But with water-gas there is no escape when once it has asserted its influence. Since the restrictions formerly imposed on the employment of water gas in certain parts of America have been removed, "gas accidents" have largely increased. The Board of Gas Commissioners of the State of Massachusetts recently presented a report, from which it appears that the deaths from this cause have increased as follows: In 1889, 2; in 1890, 10; in 1891, 12; in 1892, 18; in 1893, 25; in 1894, 33; in 1895, 26; in 1896, 50; and last year, 60. It is also a matter of interest and importance to observe that, of the deaths last year, 18 were clearly suicidal. Water gas is no doubt cheap, and it is easily made in large quantities to meet any sudden emergency; but it is a very deadly poison, apt, on the one hand, to kill the unwary who do not look after their gas fittings, and ready at all times to afford an easy and painless escape from the worries of this life to any who can afford to put a few pennies in the slot!

